# Malignant Bowel Obstruction Due to Intraluminal Metastasis of Endometrial Adenocarcinoma Located in the Sigmoid Colon After Nine Years of Follow-Up

**DOI:** 10.7759/cureus.47937

**Published:** 2023-10-29

**Authors:** Belén Matías-García, Fernando Mendoza-Moreno, Manuel Díez-Alonso, Carolina D'Angelo, Alberto Gutiérrez-Calvo

**Affiliations:** 1 General Surgery, Príncipe de Asturias Teaching Hospital, Alcalá de Henares, ESP; 2 Anatomy, Príncipe de Asturias Teaching Hospital, Alcalá de Henares, ESP

**Keywords:** immunohistochemistry, metastatic endometrial cancer, colonic metastasis, endometrial adenocarcinoma, colorectal cancer

## Abstract

Endometrial adenocarcinoma is currently the most common malignant tumor of the female genital tract. In the early stages, surgical or radiotherapy treatment offers high survival rates and excellent prognosis, although late recurrences have been described. Recurrences of endometrial adenocarcinoma are more frequent in the vaginal vault; however, implants are sometimes detected in the serosa of the colon and rectum, resulting in extrinsic compression. Here, we present the case of a 77-year-old patient with a clinical history of hysterectomy, lymphadenectomy, and double adnexectomy for endometrial adenocarcinoma (International Federation of Gynecology and Obstetrics (FIGO) Ia). Nine years after the initial treatment, she presented an endoluminal recurrence in the sigmoid colon, which is exceptional. The patient underwent surgery by performing an oncological sigmoidectomy. The immunohistochemical study revealed the tumor origin as metastasis of endometrial adenocarcinoma. The patient had a favorable postoperative period, subsequently receiving adjuvant therapy and being disease-free after 18 months of follow-up.

## Introduction

Endometrial adenocarcinoma is the most common malignant neoplasm of the female genital tract [1]. In the early stages, its treatment has a good prognosis with little recurrence both local and distant. However, the appearance of metastases in different locations years after the initial treatment has been described [2]. The digestive tract is an uncommon location, with the colon or rectum being an exceptional place. Here, we present the case report of a 77-year-old patient who underwent surgery for endometrial adenocarcinoma and, after nine years without evidence of disease, developed a stenosing endoluminal metastasis in the sigmoid colon, compatible with metastasis of endometrial adenocarcinoma.

## Case presentation

We present the case of a 77-year-old female with a medical history of hypothyroidism, high blood pressure, and dyslipidemia. Her surgical history included a mastectomy with ipsilateral axillary lymphadenectomy and chemotherapy in 1989 for breast cancer, with a subsequent recurrence requiring radiotherapy nine years later. In 2013, she underwent surgery for endometrial adenocarcinoma (pT1aN0M0, stage IA of the International Federation of Gynecology and Obstetrics (FIGO) classification), performing a hysterectomy, double adnexectomy, and pelvic lymphadenectomy, with periodic controls by gynecology service with no evidence of tumor recurrence.

**Figure 1:**
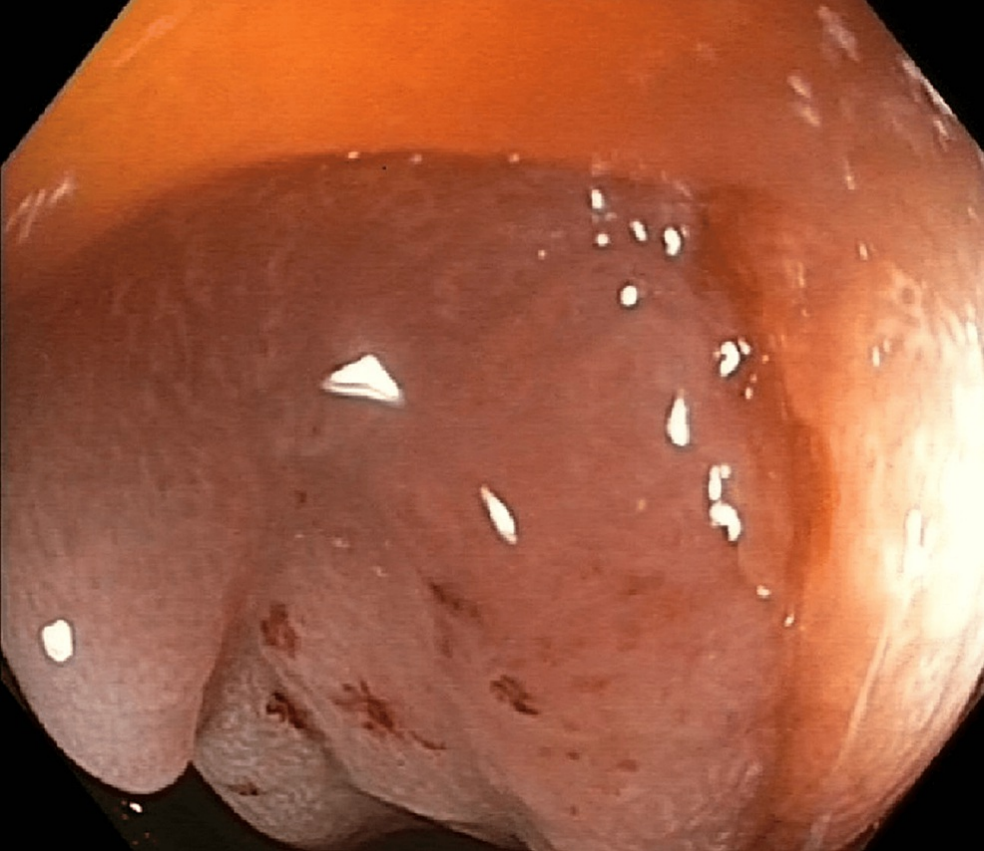
Endoscopic image for mucosal endometrial metastases located in the sigmoid colon

She underwent a colonoscopy due to symptoms of abdominal pain and constipation. In the colonoscopy, a stenosing neoplastic mass was observed 20 cm from the anal verge (Figure 1).

It was marked, and a biopsy was taken.

The histological result showed fragments of large intestine mucosa with inflammatory changes without dysplasia, focally identifying cytokeratin (CK) 7+, CK20-, and estrogen receptor (ER)+ (weak) cells, while the cytology result was positive for adenocarcinoma.

**Figure 2:**
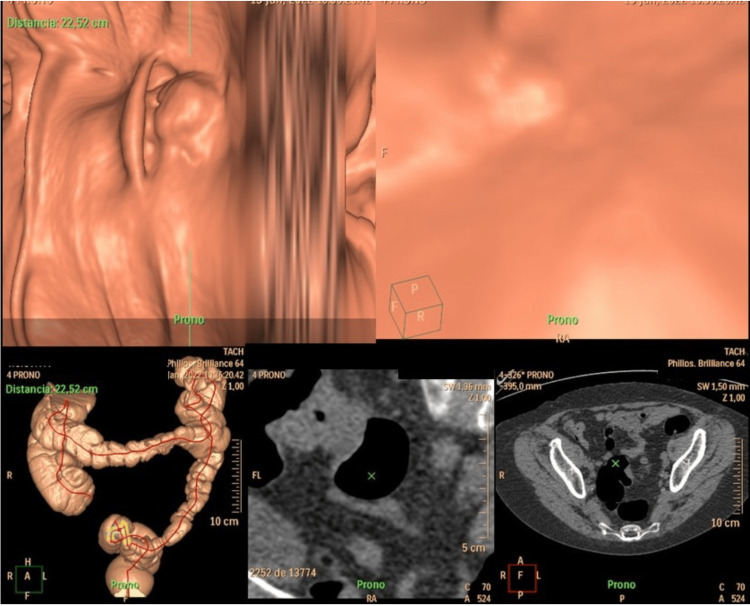
Virtual colonoscopy showing the stenotic tumor located in the sigmoid colon

The extension study was completed with a contrast-enhanced computed tomography that revealed the stenosing tumor in the sigmoid colon with locoregional lymphadenopathy (Figure 2).

No distant metastasis or peritoneal involvement was revealed. With these findings and the clinical situation of the patient, the case was presented to the multidisciplinary digestive tumors committee, and it was decided to perform a surgical intervention.

The patient underwent elective surgery in January 2022. A laparoscopic approach was performed, revealing a stenosing tumor in the rectosigmoid as well as a 2 cm peritoneal nodule located in the right iliac fossa. The liver and the rest of the peritoneal cavity did not present lesions suspicious of malignancy.

A laparoscopic sigmoidectomy was performed with descent of the splenic flexure and ligation of the inferior mesenteric vessels at the root level using XL Hem-o-Lok. In addition, the nodular lesion described in the right iliac fossa was removed. Finally, an end-to-end mechanical anastomosis was performed using CDH (Surgical Curved Intraluminal Stapler) (Ethicon Endosurgery, Cincinnati, OH) 29Fr. The patient had a favorable postoperative period and was discharged from the hospital on the fifth day.

**Figure 3:**
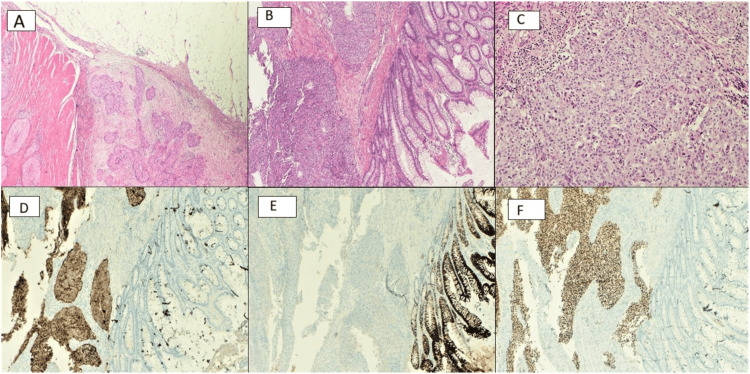
Immunohistochemical features of endometrial carcinoma/metastases: (A) endometrial metastases located in the sigmoid colon, (B) immunochemical of endometrial carcinoma/metastases, (C) original endometrial tumor, (D) immunochemical marker CK7, (E) immunochemical marker CK20, (F) immunochemical marker PAX8 CK: cytokeratin, PAX8: paired-box gene 8

The histopathological analysis of the sigmoidectomy surgical specimen showed a colorectal mucosa infiltrated by a neoplasm of epithelial origin arranged in solid nests with comedonecrosis, composed of medium-sized cells, with eosinophilic cytoplasms and nuclei with moderate atypia and pleomorphism. The neoplasia ranged from the mucosa to the perivisceral fat, reaching the proximity of the serosa of the antimesenteric border, without exceeding it. In the immunohistochemical study, the cells expressed positivity for CK7, paired-box gene 8 (PAX8), p16, and estrogens (focal and weak positive) and were negative for CK20, GATA3, mammaglobin, caudal-related homeobox gene 2 (CDX2), and progesterone. Sixteen lymph nodes were isolated, and two of them presented neoplastic infiltration (Figure 3).

The lesion located in the right iliac fossa corresponded to fibroadipose tissue infiltrated by a neoplasm of epithelial origin with a microglandular pattern, with an immunohistochemical profile similar to that described in the sigmoidectomy specimen.

These histological and immunohistochemical findings, together with the patient's personal history, were suggestive of infiltration by carcinoma of endometrial origin.

She was evaluated by the oncology service, and chemotherapy treatment consisting of six cycles was performed according to the carboplatin/paclitaxel scheme and pelvic radiotherapy according to the volumetric modulated arc therapy (VMAT) technique, with a dose of 45 Gys (1.8 Gys/day) with acceptable tolerance. The patient remains disease-free after 18 months of follow-up since the surgical intervention.

## Discussion

In 2022, approximately 65,950 new cases of endometrial adenocarcinoma were diagnosed in the United States [3]. This tumor is the most common cause of malignant neoplasia of the female genital tract. Its incidence is higher in postmenopausal females (70%) and is uncommon in females under 40 years (<5%) [1,3]. Despite this, endometrial adenocarcinoma represents the fourth most common malignant neoplasm in adult females, behind colorectal carcinoma, and the sixth cause of cancer-related death [4].

Oncological treatment in its initial stages according to the FIGO classification consists of hysterectomy, bilateral adnexectomy, and pelvic lymphadenectomy. Radiotherapy is also a therapeutic option in these stages with similar survival rates [4]. Thus, patients with stage I or II (FIGO classification) have 90% and 80% survival, respectively, at five years. Tumor recurrence in these stages is 15%, and most are detected within the first three years (75%) [3].

Although the most frequent location of metastasis from endometrial adenocarcinoma is at the lymph node level (48%), recurrence at the vagina (42%), lung (24%), and peritoneum (27%) is also common. The hematogenous or lymphatic route is responsible for them [2,5]. The location of metastases in the digestive tract is uncommon. However, involvement of the pancreas, small intestine, or rectum has been described. In these cases, the route of dissemination suggests a direct mechanism of peritoneal seeding since they are deposited in the intestinal serosa [2]. The case we present is especially atypical since the metastasis was located at the endoluminal level in the sigmoid colon.

Several risk factors have been described for the development of metastasis from endometrial adenocarcinoma, such as stage G3, myometrial invasion greater than 50%, age > 60 years, lymphovascular involvement present, and low involvement of the uterus [6]. In relation to our case, only the patient's age was a risk factor. The pathological analysis of the specimen revealed a stage IA endometrial tumor of the FIGO classification (<50% involvement of the myometrium) with complete oncological resection (R0 resection) and without the presence of the rest of the factors described.

The first reference of metastasis from an endometrial adenocarcinoma located in the rectum was made by Anstadt et al. in 2012 [7]. Later, Vogel et al. refer to the transformation of a previous focus of endometriosis into adenocarcinoma in 2013 [8]. Subsequently, metastatic involvement after a long latency period was described by Franchello et al. in 2015 [1]. Nowadays, there are three cases of metastasis at the endoluminal rectum and distal sigmoid described [2,4,9]. Our case is the first description of an endoluminal metastasis at the sigmoid colon with a stenotic pattern that developed nine years after a stage IA endometrial adenocarcinoma (FIGO classification) undergoing surgery.

Our patient presented an altered intestinal rhythm, with a predominance of constipation in relation to the endoluminal stenotic component of the tumor. The symptoms in these cases are related to the infiltrative nature of the lesion, producing a stenotic pattern and tumor growth. Other authors have described episodes of lower gastrointestinal bleeding due to tumor ulceration of the digestive tract mucosa [10].

Immunohistochemistry plays a fundamental role in the correct diagnosis of these cases. While the majority of colorectal adenocarcinomas express positivity for CDX2 and CK20, endometrial adenocarcinomas are negative for these two markers and positive for CK7, and estrogen receptor (ER). In the case of our patient, the resected sigmoidectomy specimen was positive for CK7, PAX8, p16, and estrogens (focal and weak positive) and was negative for CK20, GATA3, mammaglobin, CDX2, and progesterone. The PAX8 marker can sometimes be useful as its positivity, although weak, suggests a Müllerian origin [11,12].

The immunohistochemical result of the lesion suggests that it may be hematogenous dissemination of endometrial adenocarcinoma or malignant transformation of a previous focus of endometriosis. Endometriosis is a pathology that mainly affects females of reproductive age. However, postmenopausal endometriosis can affect up to 4% of females [13]. Therefore, malignant transformations, although infrequent, are possible [13]. The risk factors mainly related to malignant transformation are hormone replacement therapy (HRT) and the use of tamoxifen for breast cancer [13]. In the case of our patient, despite a history of breast cancer, she did not receive treatment with tamoxifen or HRT. There is also no evidence in the clinical history of a personal history of endometriosis nor a history of abdominal pain of years of evolution during fertile age or other symptoms that suggest it. In addition, the patient had a colonoscopy performed in 2012 without findings. Therefore, the possible diagnosis of endometrial adenocarcinoma secondary to malignant transformation of a previous focus of endometriosis was ruled out.

Finally, given the patient's oncological history and knowing that some malignant breast tumors can produce metastases years after the initial treatment, this diagnosis was also ruled out due to the immunohistochemistry profile.

## Conclusions

Endoluminal metastatic involvement at the colorectal location due to endometrial adenocarcinoma after a long period of latency is exceptional. Although the mechanism of dissemination has not been demonstrated, it is thought that the hematogenous route would be responsible. Its presentation as an intraluminal stenosing tumor can simulate the presentation of a colorectal carcinoma. Immunohistochemical study is essential for a correct differential diagnosis, as well as the decision of the oncological treatment and necessary subsequent follow-up.
